# Unraveling the Roles of Neuropeptides in the Chemosensation of the Root-Knot Nematode *Meloidogyne javanica*

**DOI:** 10.3390/ijms25126300

**Published:** 2024-06-07

**Authors:** Chenmi Mo, Lei Zhang

**Affiliations:** 1 Department of Botany and Plant Pathology, Purdue University, West Lafayette, IN 47907, USA; mo61@purdue.edu; 2 Department of Entomology, Purdue University, West Lafayette, IN 47907, USA

**Keywords:** neuropeptides, chemosensation, RNA sequencing, *Meloidogyne javanica*, tomato root exudate

## Abstract

The identification of novel drug targets in plant-parasitic nematodes (PPNs) is imperative due to the loss of traditional nematicides and a lack of replacements. Chemosensation, which is pivotal for PPNs in locating host roots, has become a focus in nematode behavioral research. However, its underlying molecular basis is still indistinct in such a diverse group of PPNs. To characterize genes participating in chemosensation in the Javanese root-knot nematode *Meloidogyne javanica*, RNA-sequencing of the second-stage juveniles (J2s) treated with tomato root exudate (TRE) for 1 h and 6 h was performed. Genes related to chemosensation in *M*. *javanica* mainly responded to TRE treatment at 1 h. Moreover, a gene ontology (GO) analysis underscored the significance of the neuropeptide G protein-coupled receptor signaling pathway. Consequently, the repertoire of putative neuropeptides in *M*. *javanica*, including FMRFamide-like peptides (FLPs), insulin-like peptides (ILPs), and neuropeptide-like peptides (NLPs), were outlined based on a homology analysis. The gene *Mjflp-14a*, harboring two neuropeptides, was significantly up-regulated at 1 h TRE treatment. Through peptide synthesis and J2 treatment, one of the two neuropeptides (MjFLP-14-2) was proven to influence the J2 chemotaxis towards tomato root tips. Overall, our study reinforces the potential of nematode neuropeptides as novel targets and tools for root-knot nematode control.

## 1. Introduction

Plant-parasitic nematodes (PPNs) pose a formidable threat to global agriculture, causing substantial yield losses in crops [1,2]. For decades, nematicides have served as one of the primary interventions for controlling PPNs. However, the extensive restriction on nematicides’ application and the emergence of drug resistance among nematode populations have exacerbated the impact of nematode infestations. Therefore, there is a growing recognition of searching for alternative approaches to nematode management. Biocontrol methods that exploit nematophagous fungi and bacteria and crop rotation with non-host plants represent environmentally friendly options by disturbing the life cycle and living environment of nematodes [3,4,5,6,7]. Developing crop varieties carrying natural resistance genes via crop breeding or a genetic engineering approach is also an effective strategy [8,9]. However, there are shortcomings associated with these strategies, such as economic losses, low application scope, unstable resistance, and resistance breakthroughs [10,11]. Hence, it is necessary to develop novel management approaches. Considering the special neuronal uptake system of amphids in nematodes and its function in chemosensation, the disruption of nematode behaviors using neuropeptides as targets has become an innovative route for PPN control [12,13].

Neuropeptides are one of the most ancient signaling molecules and are abundant in the phylum Nematoda [14]. In the model nematode *Caenorhabditis elegans*, there are at least 113 identified neuropeptide genes encoding over 250 putative neuropeptides [15]. Nematode neuropeptides are classified into three large groups, the FMRFamide-like peptides (FLPs), the insulin-like peptides (ILPs), and the neuropeptide-like peptides (NLPs) [15]. After being processed from precursors and post-translationally modified, bioactive neuropeptides are secreted from the nervous system and bind to their specific receptors on the cell surface [15]. Besides ILPs, which signal through receptor tyrosine kinases, the majority of FLPs and NLPs are thought to be ligands of G-protein coupled receptors (GPCRs) [16,17]. Coupled with the intricate and interconnected nature of cellular signaling pathways, neuropeptide–receptor couples have been proven to modulate various physiological processes in nematodes, such as chemosensation, locomotion, feeding, and reproduction [18,19].

Benefiting from the high conservation of neuropeptides within the phylum Nematoda and the implementation of the genome sequencing of PPNs, neuropeptide genes have been systematically analyzed and identified in several important PPNs, such as *Globodera* spp. and *Meloidogyne* spp. [20,21,22]. In order to link neuropeptides to the coordination of nematode behaviors, especially for successful parasitism in plant roots, functional studies of those identified neuropeptides were carried out primarily using RNA interference technologies. For example, the silencing of *flp-1*, *-6*, *-12*, *-14*, or *-18* in *G*. *pallida* resulted in abnormal locomotion phenotypes [23]. After soaking with the double-stranded RNA (dsRNA) of *M*. *incognita flp-14* and *flp-18*, the number of nematodes attracted by the host was significantly decreased, thereby resulting in a reduced nematode infection [24]. The knockdown of *nlp-3* and *nlp-12* in *M*. *incognita* showed a significant reduction in attraction and infection in tomato roots [25]. In addition, the use of synthetic bioactive neuropeptides is also an effective approach, with which a certain number of NLPs that impact chemosensation and host invasion was identified in *M*. *incognita* and *G*. *pallida* [26]. However, as mentioned above, the studies of neuropeptides in PPNs are currently conducted in limited species, while the information of neuropeptides in other important PPNs is still lacking.

The Javanese root-knot nematode *Meloidogyne javanica*, as one of the most concerned root-knot nematodes, is widely distributed in warm and tropical regions [2]. It establishes a permanent feeding site within the roots of a wide variety of host plants, including economically important crops. Here, we summarized the neuropeptide repertoire in *M*. *javanica* according to the annotation in the Wormbase Parasite database and performed homology-based BLAST to identify novel and missing entries that are not fully annotated. Based on the previous finding that tomato root exudate (TRE) was highly attractive to *M*. *javanica* J2s [27], the RNA-seq of *M*. *javanica* J2s treated with TRE was conducted, and the involvement of *Mjflp-14* genes in regulating chemosensation in *M*. *javanica* emerged and was further confirmed using synthetic neuropeptides.

## 2. Results

### 2.1. TRE Treatment Causes Transcriptional Changes in M. javanica

**Figure 1:**
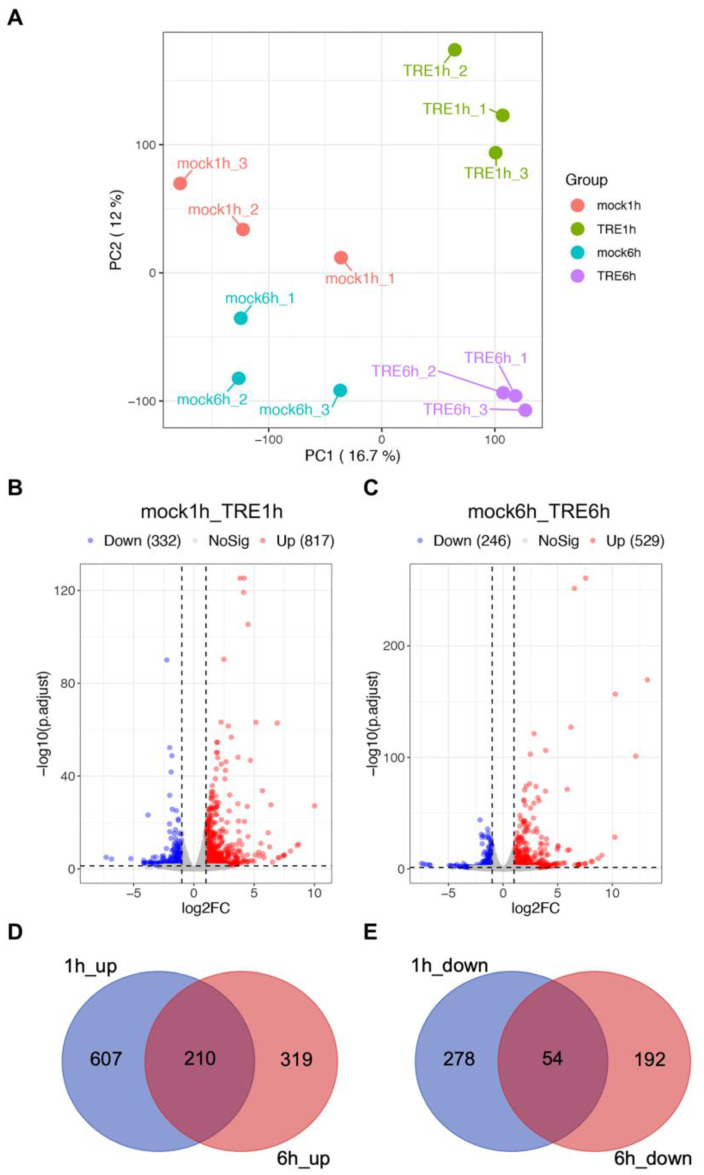
The intergroup analysis of RNA-seq data. (**A**) The PCoA analysis of the samples in RNA-seq. The fragments per kilobase million of each sample were used for drawing. (**B**,**C**) Volcano plots of differentially expressed genes (DEGs) in the comparison groups of mock1h_TRE1h (**B**) and mock6h_TRE6h (**C**). Genes with the false discovery rate < 0.05 were used to generate volcano plots, and the log2(fold change) > 1 and log_2_(fold change) < −1 were considered as up-regulated and down-regulated DEGs, respectively. (**D**,**E**) Venn diagrams of DEGs between the 1h_up and 6h_up (**D**) and 1h_down and 6h_down (**E**) groups.

To explore how *M*. *javanica* responds to tomato root exudate (TRE) treatment at the transcriptome level, 12 libraries were constructed for RNA-seq, including J2s of *M. javanica* with 1 h and 6 h mock treatments and treatments with TRE. A total of 415,047,795 raw paired reads from 12 libraries was generated, of which 409,408,914 reads survived after filtering low-quality reads and adaptors (Table 1). Filtered reads from each library were aligned to the genome of *M*. *javanica* (ASM90000394v1) using HiSAT2 with an approximate mapping rate of 94.50–96.32% (Table 1). The PCoA analysis showed independent clustering between mock and TRE treatments (Figure 1A). The R package edgeR was used to further identify the differentially expressed genes (DEGs) in the comparison groups of mock1h_TRE1h and mock6h_TRE6h. A total of 1149 DEGs were identified in the TRE1h compared to the mock1h, of which 817 and 332 genes were up-regulated and down-regulated, respectively (Figure 1B, Table S1). Few numbers of DEGs were found in the mock6h_TRE6h comparison group, with 529 genes up-regulated and 246 genes down-regulated (Figure 1C, Table S1). Only 210 up-regulated and 54 down-regulated genes were shared between the 1 h and 6 h DEGs (Figure 1D,E).

### 2.2. M. javanica Primarily Responds to TRE at 1 h

**Figure 2:**
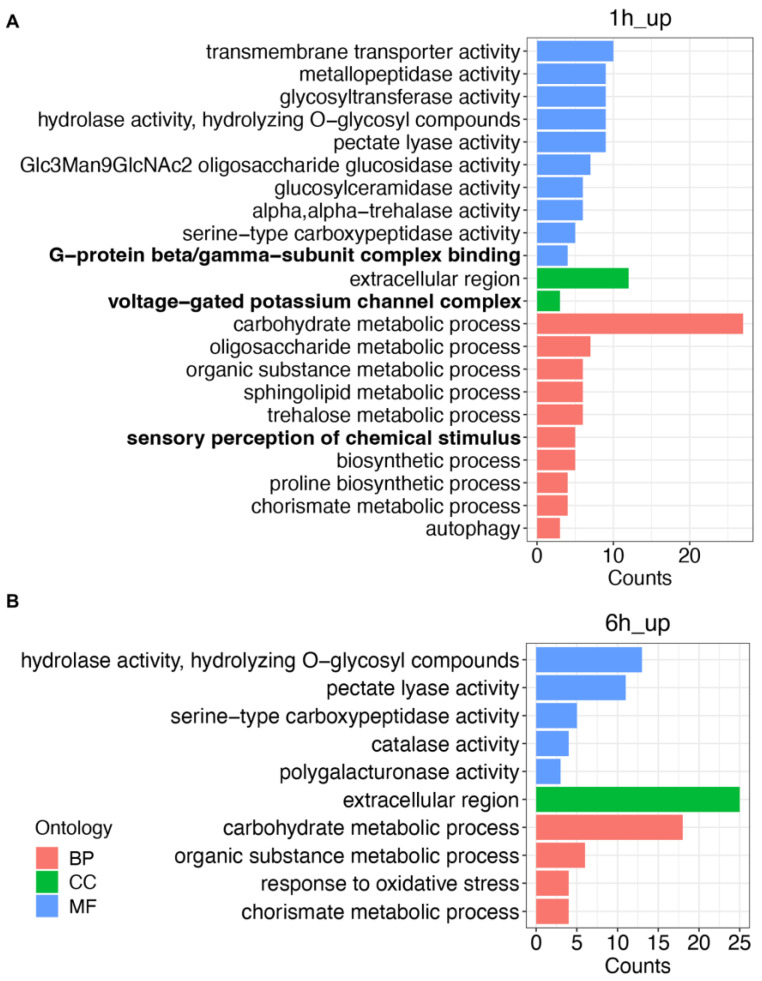
The gene ontology (GO) enrichment analysis of the DEGs in 1h_up (**A**) and 6h_up (**B**). GO terms belonging to the biological process (BP), cellular component (CC), and molecular function (MF) were displayed in red, green, and blue, respectively. GO terms related to cell signaling were bolded.

To better understand the functional classification of DEGs, especially the genes positively responding to TRE treatment, the 20 genes with the highest up-regulated expression in 1h_up and 6h_up along with their gene function description were listed in Table 2 and Table 3. Only 8 and 7 genes have a functional description in 1h_up and 6h_up, respectively. Notably, there were 3 genes, *M.Javanica_Scaff2813g025362*, *M.Javanica_Scaff28572g095259*, and *M.Javanica_Scaff4717g035993*, that were annotated as G protein-coupled receptors (GPCRs) and present only in 1h_up, and their expression levels have dropped dramatically at 6 h (Table S1). All the up-regulated DEGs in 1h_up and 6h_up were used for GO enrichment, respectively. A total of 32 GO terms were enriched in 1h_up, while only 10 GO terms were enriched in 6h_up (Table S2). Further, the top 10 GO terms selected by gene ratio were shown if the total number of GO terms belonging to molecular function (MF), cellular component (CC), or biological process (BP) exceeded 10 (Figure 2A,B). As shown in the results, a certain number of genes in 1h_up belongs to the GO terms related to chemical sensing and cellular signal transduction, including G-protein beta/gamma-subunit complex binding in MF, voltage-gated potassium channel complex in CC, and the sensory perception of the chemical stimulus in BP (Figure 2A, Table S2). Likewise, these GO terms were only found in 1h_up, while no such GO terms involved in these processes were enriched in 6h_up (Figure 2B). Taken together, *M*. *javanica* mainly responds to TRE more actively at transcriptome levels at the earlier stage (1 h) than the later stage (6 h) treatment.

### 2.3. Neuropeptide genes in M. javanica

**Figure 3:**
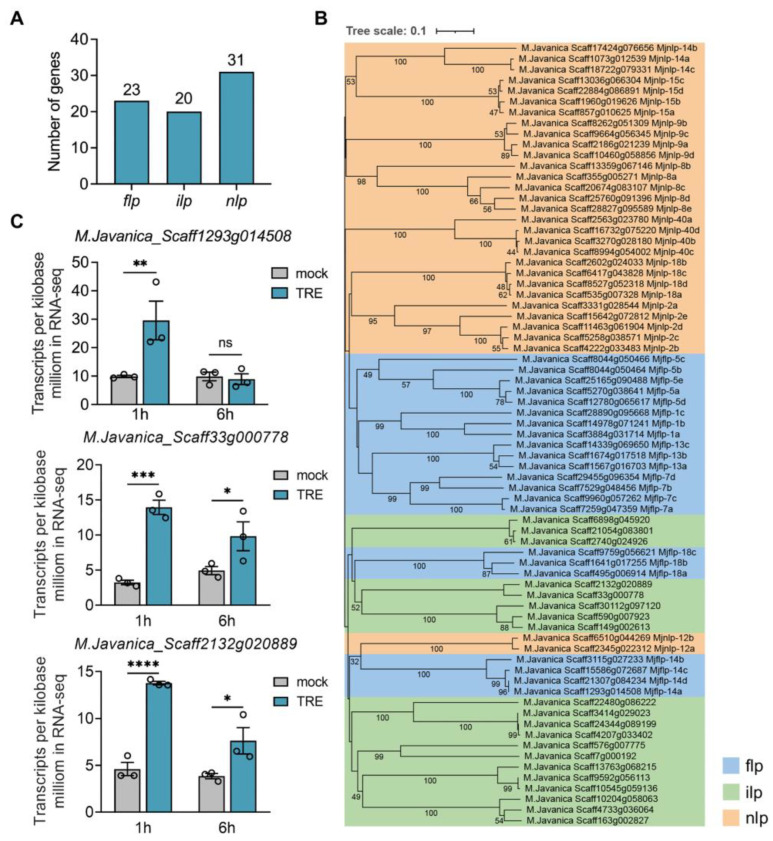
Neuropeptides in *M*. *javanica*. (**A**) The number of putative neuropeptide genes in *M*. *javanica*, including FMRFamide-like peptide (*flp*), insulin-like peptide (*ilp*), and neuropeptide-like peptide (*nlp*). (**B**) The phylogenetic analysis of all putative neuropeptide genes in *M*. *javanica*. The tree was generated by MEGA 11 using the neighbor-joining (NJ) method with 1000 bootstrap replicates. FLPs, ILPs, and NLPs were shaded by blue, green, and orange, respectively. (**C**) Transcripts per kilobase million of *M.Javanica_Scaff1293g014508*, *M.Javanica_Scaff33g000778*, and *M.Javanica_Scaff2132g020889* in RNA-seq. ns, no significance; *, *p* < 0.05; **, *p* < 0.01, ***, *p* < 0.001; ****, *p* < 0.0001.

GPCRs mediate responses to various extracellular and intracellular cues and represent the largest family of cell surface proteins in metazoan [28,29]. However, the functional overlap among GPCRs poses a challenge for the dissection of their functions. As GPCRs were found to regulate chemosensation in *C. elegans* as receptors of neuropeptides [30], it prompted us to consider whether neuropeptide genes in *M*. *javanica* also respond to TRE. Due to the lack of a summary of neuropeptides in *M*. *javanica*, the first attempt was made to identify genes encoding neuropeptides at the genome level. Through sequence alignment and a consensus analysis, a total of 74 neuropeptide genes were obtained in *M*. *javanica*, including 23 FMRFamide-like peptides (*flps*), 20 insulin-like peptides (*ilps*), and 31 other neuropeptide-like proteins (*nlps*) (Figure 3A, Table S3). All of the genes identified except *ilps* were successfully classified and named in accordance with the consensus peptide sequences of known neuropeptides in *C. elegans* (Table S3). Although these three types of genes were not fully discriminative clustering in the phylogenetic analysis, the paralogous genes were strictly clustered into the same branch (Figure 3B). Combining with the RNA-seq data, 3 of the 74 neuropeptide genes were found to be significantly up-regulated under TRE treatment. The gene *M.Javanica_Scaff1293g014508* (*Mjflp-14a*) was significantly up-regulated at 1 h of TRE treatment, and two *ilp* genes *M.Javanica_Scaff33g000778* and *M.Javanica_Scaff2132g020889* were up-regulated both at 1 h and 6 h (Figure 3C).

### 2.4. The Precursors of MjFLP-14 Neuropeptides (Pro-Neuropeptide) Contain Two Neuropeptides

**Figure 4:**
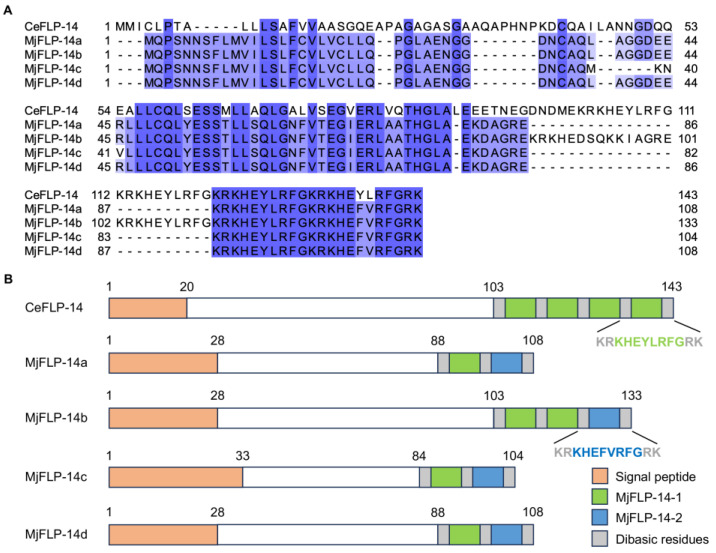
The sequence analysis of FLP-14 paralogs in *M*. *javanica* (MjFLP-14). (**A**) The sequence multiple alignment of MjFLP-14 with the FLP-14 in *C*. *elegans* (CeFLP-14). Residues were shaded with gradient colors according to the amino acid conservation. (**B**) Schematic diagram of the protein sequences of MjFLP-14 and CeFLP-14 in proportion to the length.

The pro-neuropeptide sequences of the three responsive genes were further analyzed using the homologs of *C*. *elegans* as a reference to determine the processed neuropeptides. However, no homologs of the two ILPs encoding *M.Javanica_Scaff33g000778* and *M.Javanica_Scaff2132g020889* were found in *C*. *elegans*. The four MjFLP-14 pro-neuropeptides showed sequence similarities with the FLP-14 in *C*. *elegans*, with the highest identity of MjFLP-14b reaching to 62.16% (Figure 4A). A further comparison of the FMRFamide-like domain revealed that all MjFLP-14 had two distinctly processed neuropeptides, MjFLP-14-1 (KHEYLRFG) and MjFLP-14-2 (KHEFVRFG), flanked by dibasic residues (Figure 4B). MjFLP-14-1, which shared the same sequence as the neuropeptide in CeFLP-14, was located in the front, followed by MjFLP-14-2, which has two residues different from MjFLP-14-1 (Figure 4B). In addition, unlike CeFLP-14 possessing four copies of the neuropeptides, MjFLP-14b has two copies of MjFLP-14-1 and one copy of MjFLP-14-2, and the other three sequences only have one copy of MjFLP-14-1 and MjFLP-14-2 each (Figure 4B).

### 2.5. MjFLP-14-2 Affects the Root Chemotaxis of M. javanica

**Figure 5:**
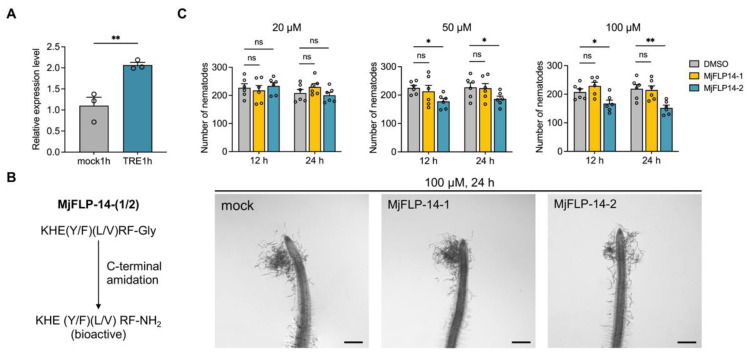
Synthetic MjFLP-14-2 affects the chemotaxis of the second juveniles (J2s) of *M*. *javanica*. (**A**) The relative expression level of *Mjflp-14a* at TRE1h compared to mock1h with RT-qPCR. (**B**) A schematic diagram of the C-terminal amidation of MjFLP-14-1 and MjFLP-14-2. (**C**) The chemotactic assay of tomato root tips by J2s of *M*. *javanica*. J2s were treated with synthetic MjFLP-14-1 or MjFLP-14-2 with different final concentrations (20, 50, and 100 μM) for 12 and 24 h. Tomato roots germinated for 7 days were placed in each well of 6-well plates with 2 mL 0.85% slurry agar mixed with 500 J2s per well, and the number of J2s attracted by tomato root tips was counted at 12 h after placing tomato roots. Dimethylsulfoxide (DMSO) solution diluted with sterile DiH_2_O was used to treat J2s as a mock treatment. Bar = 500 μm. ns, no significance; *, *p* < 0.05; **, *p* < 0.01.

The increased gene expression of *Mjflp-14a* at TRE1h was further confirmed with RT-qPCR (Figure 5A). To test whether MjFLP-14-1 and MjFLP-14-2 affect nematode behaviors, the two neuropeptides were synthesized with the functional post-translational modification of C-terminal amidation (Figure 5B). The effect of bioactive peptides on the root chemotaxis of *M*. *javanica* J2s was determined. The results showed that after being treated with MjFLP-14-2 at final concentrations of 50 and 100 μM for 12 and 24 h, the number of J2s attracted by root tips was significantly lower than that of the mock treatment, but no significant difference was found at 20 μM (Figure 5C). There is no significant difference between the mock and MjFLP-14-1 treatments (Figure 5C).

## 3. Discussion

The successful parasitism of PPNs in plant roots requires a series of behavioral coordinations, including chemosensation, locomotion, penetration, and feeding. Among these behaviors, chemosensation represents an early step in the process of host finding. Chemicals in root exudates secreted by host plants act as signals for root-knot nematodes, attracting them towards the root tip region of host plants [27,31,32,33,34]. Therefore, understanding the interplay between root exudates and PPNs is crucial for developing strategies to manage nematodes. We designed an experiment to treat *M*. *javanica* with collected tomato root exudates alone and then used them for RNA-seq, by which it is possible to exclude the effects of physical interaction with plant roots and focus on how the root exudates affect nematodes. The feasibility of this treatment was confirmed with a GO enrichment analysis of the DEGs. There are GO terms related to signal sensing and transduction pathways enriched in the 1h_up as well as pectate lyase activity, which is found both in the 1h_up and 6h_up (Figure 2A,B). These results indicate that treatment with tomato root exudate alone can successfully induce a response in nematodes, as it does around the roots.

Neuropeptides and components of the signal pathways where they are involved have become intriguing targets for PPN control [12]. The present study identified a total of 74 neuropeptide genes in *M*. *javanica*, whose genome is proposed to be tetraploid [21]. Indeed, we found that many groups of neuropeptides possess four paralogous genes; however, a certain number of groups had only three paralogous genes (Figure 3B). This may be due to loci loss during polyploidization or incomplete sequence assembly and annotation. The FLPs and NLPs in *M*. *javanica* showed high sequence conservation and were effortlessly assigned into the same groups as in *C*. *elegans*. However, although the insulin-like domain was present in all ILPs in *M*. *javanica*, no homologs of *C*. *elegans* were found, even for the INS-1, -17, and -18 that are found to be widely distributed within nematodes, including plant-parasitic, human-parasitic, animal-parasitic, EPN, and free-living nematodes [35]. Therefore, they were not assigned and named by homology.

It is not yet known how fast the nematodes respond to TRE after sensing it, so two time points (1 h and 6 h) of mock and TRE treatments were chosen for the RNA-seq. In the DEGs list of RNA-seq, only 3 of 74 neuropeptide genes, *Mjflp-14a* and two *Mjilp* genes, were included. This is explainable because PPNs are able to perceive root exudates and thereby tailor gene expression for what is currently needed for host parasitism [36]. The two *ilp* genes exhibited a similar expression pattern, as they are paralogs. However, of the four *Mjflp-14* genes, only *Mjflp-14a* was significantly up-regulated. The contingency derived from the experiment or RNA sequencing was first excluded because the abundance of *Mjflp-14a* gene expression has increased dramatically at 1 h of TRE treatment (Figure 3C). A possible reason is that the expression of *Mjflp-14a* is under a special regulatory mechanism in the process of perceiving the host. In addition to comparing the mock1h_TRE1h and mock6h_TRE6h groups, the DEG analysis was also performed between mock1h and mock6h (mock1h_mock6h). Only 41 and 113 genes were significantly up-regulated and down-regulated, respectively. No specific GO terms of biological processes were enriched within these DEGs, suggesting a stable status of J2s in the short time scale. Moreover, the consistent expression level of *Mjflp-14a* in both mock1h and mock6h suggests a stable expression of the gene in the absence of TRE.

FLP-14 is a highly conserved and abundant neuropeptide amongst nematodes [37,38]. It was found to be expressed in neurons of *C*. *elegans* and the SMB-like neurons of the PPN *M*. *incognita*, implying its role in regulating locomotion and sensory processing [38,39]. In PPNs, the significance of *flp-14* for the infection and development of *M*. *incognita* was demonstrated through the RNAi of the gene in vitro and in planta [24,40]. The similar phenotype of *flp-14* silencing was also found in *M*. *graminicola* [41]. These studies demonstrated a deficient expression level of *flp-14*-affected nematode behaviors. Oppositely, our study used synthetic MjFLP-14-2 to treat nematodes, which is equivalent to excessive levels and also obtained similar results of negatively regulating chemotaxis. This can be explained by the precise regulatory requirement of *flp-14* levels in *C*. *elegans*, pointing out that abnormal levels of *flp-14* and its receptor of the same neuropeptide pathway, whether excessive or deficient, disrupt normal functions, leading to significant behavioral defects [39]. Thus, our study indicated a conserved function of *flp-14* amongst nematodes. Unlike the FLP-14 in *C*. *elegans*, which was processed into one active neuropeptide, MjFLP-14 is able to be processed into two similar but different neuropeptides (Figure 4B). Nevertheless, only MjFLP-14-2 showed negatively regulation in chemotaxis of *M*. *javanica* via treating the J2s with a synthetic peptide. Unlike MjFLP-14-1, the MjFLP-14-2 peptide sequence is slightly different from FLP-14 in *C. elegans*, indicating variations of activity or the function of similar neuropeptides among distinct nematode species. The role of MjFLP-14-2 in negatively affecting the host finding of *M*. *javanica* and potentially other RKNs could designate it as a promising target for interference strategies aimed at preventing RKN infections in crops.

## 4. Materials and Methods

### 4.1. Nematode Propagation

The root-knot nematode *Meloidogyne javanica* was cultured on tomato plants (cv. *Rutgers*) under greenhouse conditions (23–26 °C, 16/8 h day/night period). Eggs of *M*. *javanica* were collected by treating the infected tomato roots with 0.8% NaClO for 5 min followed by continuous washing through a series of 250, 45, 25 μm mesh sieves. Freshly prepared eggs were used for the second-stage juveniles (J2s) hatching in 0.1% (*v*/*v*) plant preservative mixture (PPM) solution.

### 4.2. Tomato Root Exudate (TRE) Collection

TRE was collected as described with modifications [27]. Rutgers tomato seeds were germinated on autoclaved germination paper soaked in autoclaved deionized water (DiH_2_O) in petri dish for 10 days. An amount of 70 seedlings were rolled together such that the terminal 10 mm of roots were submerged in 8 mL of autoclaved DiH_2_O in a 100 mL beaker. The 100 mL beaker was placed in a 500 mL beaker with a wet Kimwipe^®^ tissue (Kimtech Science™, Dallas, TX, USA) at the bottom, and the whole setup was covered with a sheet parafilm with a few pin holes for ventilation. The setup was kept at 25 °C in the dark. After 24 h, the liquid in the 100 mL beaker was collected and passed through a 0.22 μm filter. This TRE can be used immediately or stored at −20 °C.

### 4.3. Sample Collection for RNA-Seq

Freshly hatched *M*. *javanica* J2s were concentrated by natural settling down for 1 h, and the concentration of J2s was determined. An amount of 1.2 mL of TRE was added to each well of the 12-well plate, and then, 2000 J2s were added and mixed well. The plates were kept at 25 °C in the dark and gently shaken every 20 min. The solutions were transferred to 1.5 mL low-retention tubes after 1 h and 6 h and centrifuged at 7000 rpm for 1 min. The supernatant was carefully removed and then snap frozen in liquid nitrogen before being stored at -80 °C. Autoclaved DiH_2_O instead of TRE was used as mock treatment.

### 4.4. RNA Extraction, Library Preparation, and RNA-Seq

Total RNA of each sample was extracted using the PureLink^®^ RNA Mini Kit (Invitrogen™, Waltham, MA, USA) following the manual with three technical replicates. The quality and quantity of RNA from each sample were determined by the Agilent 5400 system in Genomics Core at Purdue University, and the RNA integrity numbers (RINs) of all samples were larger than 9.

### 4.5. Analysis of RNA-Seq Data

Raw reads from each sample were trimmed to filter low-quality bases at both 5′- and 3′- ends, length below 50 bp, and the contaminated adapters using Trimmomatic 0.38 [42]. The clean reads generated were mapped to the reference genome of *M*. *javanica*, downloaded from the Wormbase Parasite (https://parasite.wormbase.org/Meloidogyne_javanica_prjeb8714/Info/Index, accessed on 16 October 2023) using HiSAT2 software (v 2.2.1) [43]. SAM files were converted into BAM files and sorted using Samtools-1.15. *featureCounts* was employed to count mapped reads, taking exon as a feature [44]. Differential gene expressions between control and treatment were determined on the counts using the Bioconductor software edgeR v 3.38.4 [45]. Genes were considered to be significant DEGs when the absolute value of log_2_(fold change) > 1 and the false discovery rate (FDR) < 0.05. The GO enrichment of DEGs was implemented using the *enricher* function provided by the clusterProfiler R package v 4.4.4 [46]. The GO annotation file of *M*. *javanica* was downloaded from BioMart in the Wormbase Parasite database and used for enrichment analysis. GO terms with adjusted *p-value* < 0.05 were considered significantly enriched GO terms.

### 4.6. BLAST Searches for Neuropeptide Genes in M. javanica

Two methods were combined to identify the neuropeptide genes in *M*. *javanica*. For the *flp* and *nlp* genes with known sequence homology between *C*. *elegans* and *M*. *javanica* [14,26,47], the propeptide sequences of FLPs and NLPs in *C*. *elegans* were successively used as queries to perform BLASTP search against the genome of *M*. *javanica* (PRJEB8714) on the Wormbase Parasite server (https://parasite.wormbase.org/Multi/Tools/Blast, accessed on 24 October 2023) with default setting. For the *ilp* genes lacking homology information, the function of *display all genes with this domain* on the Wormbase Parasite was used to obtain all genes harboring the domain of insulin-like superfamily (InterPro, IPR036438) in *M*. *javanica*. The FMRFamide-related peptide-like domain (InterPro, IPR002544) was also applied to this function search to generate a new output of *flp* genes, which was merged with the previous output of *flp* genes after removing duplicates. Then, the resulting outputs of these three types of genes were used as queries again to perform BLASTP against the *M*. *javanica* genome to search for missing paralogous genes. Each group of paralogous genes was named alphabetically according to the last 6 digits of the gene ID from smallest to largest. To obtain the conservation of all neuropeptides in *M*. *javanica* with other nematodes, BLASTP search was performed using the amino acid sequences of neuropeptides to search against the genome of *C*. *elegans* (PRJEB13758), *M*. *incognita* (PRJEB8714), *M*. *hapla* (PRJEB29083), *M*. *enterolobii* (PRJEB36431), *M*. *arenaria* (PRJEB8714), *M*. *chitwoodi* (PRJNA666745), and *Heterodera glycines* (PRJNA381081).

### 4.7. Post-BLAST Analysis

All BLAST-generated genes were manually inspected for consensus peptide sequences flanked by mono/dibasic cleavage sites, and the sequences that showed sequence homology but did not contain consensus peptide sequences were excluded. The remaining genes were designated to the cluster of neuropeptides in *C*. *elegans* based on sequence homology. Signal peptides were predicted by SignalP 6.0 [48], but the sequences lacking a predicted signal peptide did not exclude neuropeptide designation.

### 4.8. Phylogenetic Tree Construction

Multiple sequence alignment of the neuropeptide genes in *M*. *javanica* was carried out using the MUSCLE program embedded in the MEGA 11 software (v 11.0.9) with default setting [49]. The result of alignment was used as an input to construct the tree using the neighbor-joining method based on the poisson distribution. Interactive Tree of Life server (https://itol.embl.de/, 7 December 2023) was employed to annotate the tree.

### 4.9. Reverse Transcription-Quantitative Polymerase Chain Reaction (RT-qPCR)

The total RNA was treated with the TURBO DNA-free™ Kit (Invitrogen™, Waltham, MA, USA) and used to generate the first strand of cDNA using the RevertAid First Strand cDNA Synthesis Kit (Thermo Scientific, Waltham, MA, USA). Gene expression abundance of *Mjflp-14a* was analyzed using the Bio-Rad CFX96 Real Time System using the primers (forward: 5′-TCTTCTACACTTCTGTCTCAATTAGG-3′ and reverse: 5′-AACGCAAATACTCATGCTTTCTC-3′). Gene *GAPDH* in *M*. *javanica* were used as endogenous reference genes using the primers (forward: 5′-CGTGCAGCGGTTGAGAAGGA-3′ and reverse: 5′-ACGTCCGTGGGTAGAATCAT-3′). The fold changes of gene expression were calculated versus the control by the 2^−△△Ct^ method.

### 4.10. Peptide Synthesis and Root Attraction Assay

Two predicted FLP-14 peptides in *M*. *javanica* were synthesized at GenScript (Piscataway, NJ, USA) with a modification of C-terminal amidation and dissolved in DMSO to make a 100 mM stock. Rutgers tomato seeds were germinated on autoclaved germination paper soaked in autoclaved deionized water (DiH_2_O) in petri dish for 7 days. On the 6th day of germination, freshly hatched J2s of *M*. *javanica* were incubated in 500 μL of each peptide solution at a final concentration of 20, 50, and 100 μM in 1.7 mL low-retention tubes for 12 and 24 h. An amount of 2 mL of 0.85% (*w*/*v*) water agar was added to each well of the 6-well plate, and then, 200 μL of 500 treated J2s was added and mixed well. One germinated seedling was placed in each well with the root tips submerged in the water agar. The plates were placed at 25 °C in the dark for 12 h, and the number of J2s attracted by root tips was counted. An equal volume of DMSO instead of peptide was used as mock treatment to go through the same steps.

### 4.11. Statistics Analysis

Data were presented as mean ± standard error of mean (SEM), and all statistical tests were performed using GraphPad Prism 10 (v 10.1.1, GraphPad Software, La Jolla, CA, USA). Comparisons between groups were performed using unpaired two-tailed parametric *t* test. A *p*-value < 0.05 was considered statistically significant.

## Figures and Tables

**Table 1:** Statistics of RNA-seq.

Sample	Raw Data	Clean Data	Survival Percentage (%)	Mapping Rate (%)
mock1h_1	38,356,574	37,765,286	98.46	95.21
mock1h_2	35,490,768	34,994,387	98.60	95.85
mock1h_3	32,613,765	32,155,106	98.59	96.32
TRE1h_1	29,664,312	29,282,765	98.71	95.21
TRE1h_2	34,020,431	33,620,422	98.82	95.87
TRE1h_3	31,189,106	30,799,201	98.75	95.81
mock6h_1	32,607,581	32,126,436	98.52	94.50
mock6h_2	34,724,144	34,277,033	98.71	95.69
mock6h_3	42,798,243	42,222,837	98.66	95.69
TRE6h_1	33,362,074	32,944,845	98.75	95.26
TRE6h_2	36,609,225	36,141,861	98.72	95.11
TRE6h_3	33,611,572	33,078,735	98.41	95.22
Total	415,047,795	409,408,914		

**Table 2:** The description of the top 20 up-regulated DEGs (mock1h_TRE1h).

	Gene ID	log_2_FC	Description
1	M.Javanica_Scaff1717g017816	10.01	Zinc finger nuclear hormone receptor-type
2	M.Javanica_Scaff8002g050303	8.70	-
3	M.Javanica_Scaff13615g067812	8.55	Zinc finger nuclear hormone receptor-type
4	M.Javanica_Scaff2813g025362	8.01	7TM GPCR serpentine receptor class g (Srg)
5	M.Javanica_Scaff275g004293	7.51	-
6	M.Javanica_Scaff28572g095259	7.48	7TM GPCR serpentine receptor class d (Srd)
7	M.Javanica_Scaff7668g048992	7.41	-
8	M.Javanica_Scaff11760g062770	7.09	-
9	M.Javanica_Scaff14283g069509	6.97	-
10	M.Javanica_Scaff4004g032378	6.91	-
11	M.Javanica_Scaff13439g067345	6.51	Zinc finger nuclear hormone receptor-type
12	M.Javanica_Scaff1298g014556	6.39	Pectin lyase fold/virulence factor
13	M.Javanica_Scaff21028g083757	6.29	-
14	M.Javanica_Scaff275g004289	6.16	-
15	M.Javanica_Scaff4732g036058	5.81	-
16	M.Javanica_Scaff4717g035993	5.72	7TM GPCR chemoreceptor (Srsx)
17	M.Javanica_Scaff22685g086570	5.70	-
18	M.Javanica_Scaff22685g086569	5.56	-
19	M.Javanica_Scaff5658g040440	5.43	CAP superfamily
20	M.Javanica_Scaff28396g095016	5.36	-

**Table 3:** The description of the top 20 up-regulated DEGs (mock6h_TRE6h).

	Gene ID	log_2_FC	Description
1	M.Javanica_Scaff4047g032589	13.20	-
2	M.Javanica_Scaff1588g016853	12.14	-
3	M.Javanica_Scaff1298g014556	10.27	Pectin lyase fold/virulence factor
4	M.Javanica_Scaff1588g016854	10.22	-
5	M.Javanica_Scaff13615g067812	9.05	Zinc finger nuclear hormone receptor-type
6	M.Javanica_Scaff2019g020060	8.71	-
7	M.Javanica_Scaff14881g071003	8.18	-
8	M.Javanica_Scaff8002g050303	8.05	-
9	M.Javanica_Scaff4004g032378	7.55	-
10	M.Javanica_Scaff110g002058	7.53	-
11	M.Javanica_Scaff7797g049511	7.50	-
12	M.Javanica_Scaff12043g063606	7.13	-
13	M.Javanica_Scaff1896g019155	6.95	-
14	M.Javanica_Scaff5267g038619	6.92	-
15	M.Javanica_Scaff7479g048256	6.92	-
16	M.Javanica_Scaff17664g077171	6.87	Ribonuclease H-like superfamily
17	M.Javanica_Scaff410g005950	6.84	NAD(P)-binding domain superfamily
18	M.Javanica_Scaff4857g036668	6.58	Zona pellucida domain
19	M.Javanica_Scaff13439g067345	6.52	Zinc finger nuclear hormone receptor-type
20	M.Javanica_Scaff879g010834	6.21	Pectin lyase fold/virulence factor

## Data Availability

The raw data in RNA-seq for this study were submitted to the National Center for Biotechnology Information (NCBI) under BioProject number PRJNA1098081.

## Supplementary Materials

The following supporting information can be downloaded at https://www.mdpi.com/article/10.3390/ijms25126300/s1.
